# A Developmental Surveillance Score for Quantitative Monitoring of Early Childhood Milestone Attainment: Algorithm Development and Validation

**DOI:** 10.2196/47315

**Published:** 2023-08-18

**Authors:** Yonatan Bilu, Guy Amit, Tamar Sudry, Pinchas Akiva, Meytal Avgil Tsadok, Deena R Zimmerman, Ravit Baruch, Yair Sadaka

**Affiliations:** 1 KI Research Institute Kfar Malal Israel; 2 Neuro-Developmental Research Center Mental Health Institute Be’er-Sheva Israel; 3 TIMNA Inititative Big Data Platform Israel Ministry of Health Jerusalem Israel; 4 Public Health Services Israel Ministry of Health Jerusalem Israel; 5 Faculty of Health Sciences Ben-Gurion University of the Negev Be’er-Sheva Israel

**Keywords:** child development, risk scores, scoring methods, language delay, motor skills delay, developmental, surveillance, developmental delays, developmental milestones, young children, intervention, child

## Abstract

**Background:**

Developmental surveillance, conducted routinely worldwide, is fundamental for timely identification of children at risk of developmental delays. It is typically executed by assessing age-appropriate milestone attainment and applying clinical judgment during health supervision visits. Unlike developmental screening and evaluation tools, surveillance typically lacks standardized quantitative measures, and consequently, its interpretation is often qualitative and subjective.

**Objective:**

Herein, we suggested a novel method for aggregating developmental surveillance assessments into a single score that coherently depicts and monitors child development. We described the procedure for calculating the score and demonstrated its ability to effectively capture known population-level associations. Additionally, we showed that the score can be used to describe longitudinal patterns of development that may facilitate tracking and classifying developmental trajectories of children.

**Methods:**

We described the Developmental Surveillance Score (DSS), a simple-to-use tool that quantifies the age-dependent severity level of a failure at attaining developmental milestones based on the recently introduced Israeli developmental surveillance program. We evaluated the DSS using a nationwide cohort of >1 million Israeli children from birth to 36 months of age, assessed between July 1, 2014, and September 1, 2021. We measured the score’s ability to capture known associations between developmental delays and characteristics of the mother and child. Additionally, we computed series of the DSS in consecutive visits to describe a child’s longitudinal development and applied cluster analysis to identify distinct patterns of these developmental trajectories.

**Results:**

The analyzed cohort included 1,130,005 children. The evaluation of the DSS on subpopulations of the cohort, stratified by known risk factors of developmental delays, revealed expected relations between developmental delay and characteristics of the child and mother, including demographics and obstetrics-related variables. On average, the score was worse for preterm children compared to full-term children and for male children compared to female children, and it was correspondingly worse for lower levels of maternal education. The trajectories of scores in 6 consecutive visits were available for 294,000 children. The clustering of these trajectories revealed 3 main types of developmental patterns that are consistent with clinical experience: children who successfully attain milestones, children who initially tend to fail but improve over time, and children whose failures tend to increase over time.

**Conclusions:**

The suggested score is straightforward to compute in its basic form and can be easily implemented as a web-based tool in its more elaborate form. It highlights known and novel relations between developmental delay and characteristics of the mother and child, demonstrating its potential usefulness for surveillance and research. Additionally, it can monitor the developmental trajectory of a child and characterize it. Future work is needed to calibrate the score vis-a-vis other screening tools, validate it worldwide, and integrate it into the clinical workflow of developmental surveillance.

## Introduction

With growing awareness to the high prevalence of developmental, behavioral, or social delay among young children, and the importance of early intervention to mitigate this risk [1-5], many international organizations have recommended routine developmental surveillance for all children [2,6,7]. This process is typically conducted by evaluating the children’s ability to attain a battery of age-appropriate milestones at routine clinic visits during the first few years of their life [2]. Interpreting the results of such evaluations is not straightforward. For a specific milestone, one can establish the population’s age-dependent norms of attaining the milestone and use them to assess the level of concern in case a child fails to attain it, similar to the way physical growth measures are monitored [8-10]. However, unlike physical growth norms, which are continuous and whose trajectories over time are readily understood, success or failure at attaining a developmental milestone is a binary measure, and it is not obvious how to integrate the results of multiple different milestones across several developmental domains to quantitatively monitor and assess a child’s development over time.

The assessment of child development can be done at varying level of details using 3 different types of tools: surveillance (or monitoring), screening, and evaluation. Developmental surveillance is based on milestone attainment checklists and is used worldwide by pediatricians and health care providers at routine encounters, as well as by educators and parents. Screening requires a more formal and elaborated assessment, typically done by caregivers or health care professionals at specific ages. Finally, developmental evaluation is an in-depth examination, typically done by a trained specialist, which aims to provide a formal diagnosis of the child. Importantly, surveillance is based on developmental norms, whereas screening tools are validated against a “gold standard” obtained from evaluation.

A commonly used screening tool is the Denver II Screening Tool [8,11], where the outcome is either “normal” or “suspicious,” based on how many milestones were failed and the general rate of failure for them. A common alternative is the Ages & Stages Questionnaires (ASQ-3) [12] screening tool, where caregivers select 1 of 3 answers for an array of questions, and the total score identifies the child’s development as being “on schedule,” requiring “learning activities and monitor,” or needing “further assessment.” Both of these screening tools take about 20 minutes to administer, depending on the age of the child and the experience of the person administering them. A widely used developmental evaluation tool is the Bayley Scales of Infant and Toddler Development [13], which typically takes 30-70 minutes to complete and yields a numerical score for each developmental category, as well as an estimate for a child’s developmental age—that is, at what age do neurotypical children exhibit a similar level of milestone attainment.

Previous work [14] has attempted to combine and standardize the results of 12 commonly used screening and evaluation tools into a single metric. However, doing so for surveillance tools is more challenging. There is a lack of standardization at this level of assessment, and the quantification of developmental surveillance assessments has not been previously suggested. At best, surveillance tools are calibrated using real-world data to determine the rate of milestone attainment at different ages [9] and then administered accordingly.

In this work, we suggested a relatively simple new methodology for translating a milestone-based developmental surveillance scale into a single score, denoted as the Developmental Surveillance Score (DSS), that conveys a child’s developmental status during a specified time period. Based on data from a national developmental surveillance program in Israel, we demonstrated that this score consistently captures known associations between the development and characteristics of the mother and child. Moreover, the score can be used to reveal and explore new associations, which may further improve our understanding of the factors that impact developmental delay. Finally, the score can be used to track individual children longitudinally, by describing the trajectory of their development over time. We showed that by clustering these trajectories, we can identify several typical patterns of development.

The focus of this work was on defining a straightforward surveillance score (in the sense that computing it as part of the surveillance workflow adds essentially no overhead over the current practice) and establishing its coherence and potential usefulness. Further work is required to refine this score, validate it using various data sets internationally, and derive from it explicit protocols.

## Methods

### Developmental Surveillance in Israel

Developmental surveillance (from birth to 6 years of age) in Israel is performed routinely (and free of charge) according to national standards by trained public health nurses in approximately 1000 maternal child health clinics (MCHCs). The collected data of approximately 70% of the Israeli population of this age group are documented in a single common database managed by the Israeli Ministry of Health. The developmental assessments include 59 milestones across 4 domains: personal-social, language, fine motor, and gross motor [9].

Parents are instructed to visit the MCHC after hospital discharge and then at ages of 1, 2, 4, 6, 9, 12, 18, 24, 36, 48, and 60 months. At each visit, a predefined group of age-related milestones is evaluated, according to the expected development at that age (denoted “age step”). Children may also be evaluated on milestones of a previous age step, in cases of a missed visit or a failure to attain milestones at the preceding visit.

The child’s ability to attain each milestone is reported as observed in the clinic; although in cases of difficult attainments, this may by documented according to a parent’s report. If the evaluated milestone was not attained by neither observation nor parental report, it is documented as unattained.

### Study Cohort

This study included all children born between July 1, 2014, and September 1, 2021, who were followed at the MCHCs and had at least one developmental evaluation recorded during the study period. In most of the analyses, we excluded children born preterm (gestational age of <37 weeks)—the one exception is the analysis of gestational age. Additionally, children with missing gestational age were excluded, as well as visits without developmental data or without the child’s age. The final cohort included 1,130,005 children in total, with 1,052,905 of them born on-term.

### DSS Definition

Sudry et al [9] have recently introduced the Tipat Halav Israel Surveillance (THIS) developmental scale, a data-driven developmental scale comprising curves of attainment rate by age for each of the 59 milestones evaluated in the Israeli developmental surveillance program (the scale can be downloaded from [15]). Broadly, when a child fails to attain a milestone, the THIS developmental scale categorizes the severity of this failure into 1 of 4 categories, depending on how often children of the same age fail to attain this milestone. Accordingly, in this study, we defined the Discrete Milestone Attainment Score (DMAS) for a failed milestone as the numerical order of the failure severity: a score of 1, 2, 3, or 4 is assigned for failure occurring when <75%, 75% to 90%, 90% to 95%, or >95% of the children at the same age attain this milestone, respectively. For an attained milestone, the DMAS value is 0. If an milestones is attempted multiple times, it will be scored separately each time it is attempted. The total score for a set of milestones is the average DMAS over all milestones of all developmental domains.

More formally, for each milestone, the age thresholds for attainment by 75%, 90%, and 95% of the children were calculated [9]; we denoted these age thresholds for milestone *t* by *t_75_*, *t_90_*, and *t_95_*, respectively, and considered the 4 consecutive age brackets they define:

b_1_ = [t_0_, t_75_], b_2_ = (t_75_, t_90_], b_3_ = (t_90_, t_95_], b_4_ = (t_95_, t_100_]

where *t_0_* and *t_100_* are the minimal and maximal ages at which the milestone *t* is assessed, respectively.

For a milestone *t* evaluated at age *a*, we defined *i* such that *a* is in the bracket *b_i_* (*i* indicates the severity of failure):



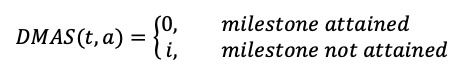



To avoid noncontinuity, we extended the above definition into a Linearized Milestone Attainment Score (LMAS), using a *c* function as follows:

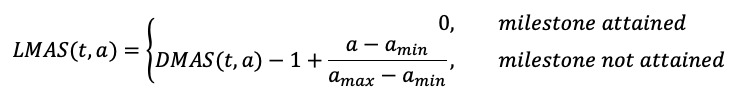



where *a_min_* and *a_max_* are the low and high ends of *b_i_*, respectively.

**Figure 1:**
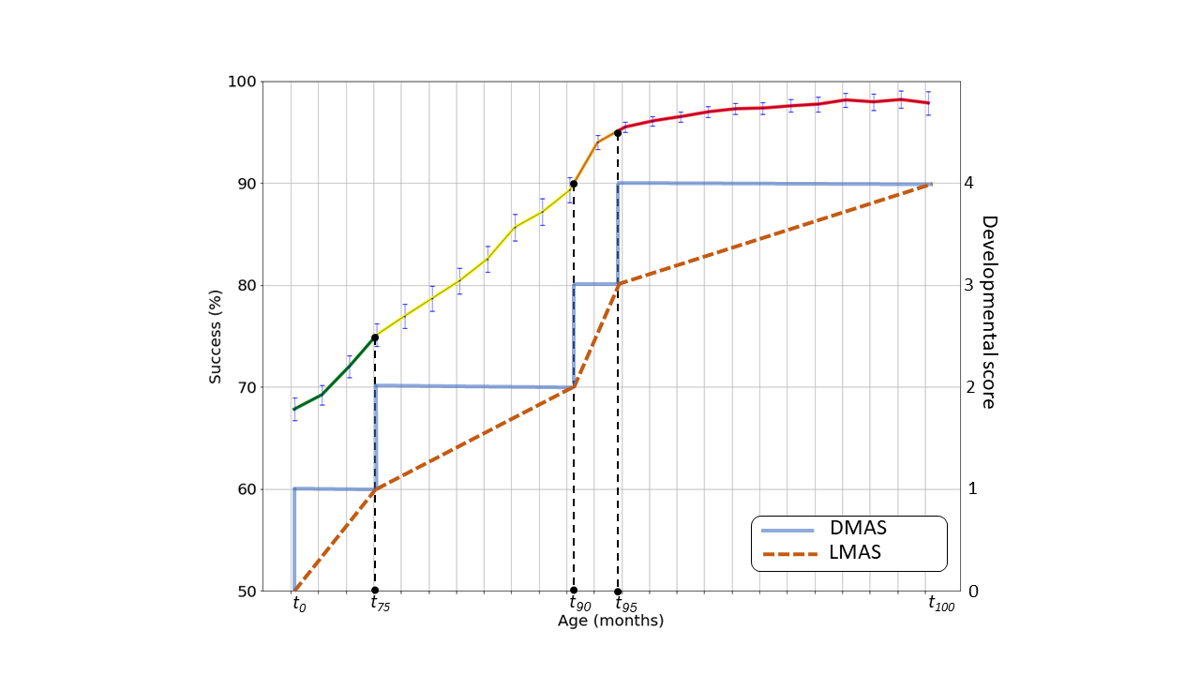
A schematic depiction of the Discrete Milestone Attainment Score (DMAS) and the Linearized Milestone Attainment Score (LMAS) computed from the trend of milestone attainment rate versus the child’s age.

The definitions of DMAS and LMAS are graphically illustrated in Figure 1. In the remainder of this paper, we used the LMAS version of the score, unless otherwise noted. In practice, deciding which of the 2 to use depends on the use case. DMAS is straightforward to compute from the THIS scale, whereas LMAS offers finer resolution.

For a set of milestones *T*, we defined the developmental surveillance score *DSS(T)* as the average of the individual milestone attainment scores:



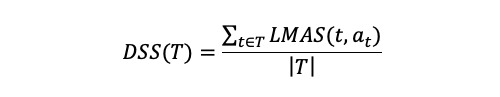



where *a_t_* is the age at which milestone *t* was assessed. See Multimedia Appendix 1 for a concrete example of computing the score.

The set of milestones used for calculating the DSS can be defined by the evaluation period and by the types of developmental domains. For example, when computing the fine-motor score for a child during the first year of life, we computed the score for each fine-motor milestone attempted by the child during this period and then the average of the scores. In particular, if a milestone was attempted multiple times during this period, all attempts were used for the calculation of the score. Determining the evaluation period is a delicate point, which depends on the DSS application. Herein, we considered a broad period of 1 year in the subpopulation analysis and visits during each MCHC-determined age bracket (typically, a single visit) when analyzing developmental trajectories.

In this study, we aggregated personal-social milestones with language milestones, denoting them as “language-social” milestones. This was motivated by the relatively small number of milestones in the social domain and the interdependence of development in these 2 domains.

In Multimedia Appendix 1, we described an alternative score definition, the q-score, which is motivated by the notion of developmental quotient and is based on a more formal statistical approach. As described there, these 2 approaches lead to a similar ranking of children according to the quantified developmental delay, that is, when asking for which of 2 given children there is a greater concern for developmental delay, the 2 approaches tend to give the same answer.

### Associations Between Mother and Child Characteristics and the Developmental Score

We examined the relations between the DSS and the characteristics of the mother and child. The children’s characteristics included sex, gestational age at birth, birth weight, birth order, and records of an existing developmental tracking.

When analyzing gestational age, we partitioned preterm births to extremely preterm (less than 27 weeks), very preterm (27-31 weeks), and late preterm (32-36 weeks) [16]. This was the only analysis that included preterm children.

Characteristics of mothers included age at delivery; level of education; and the result of postpartum depression (PPD) evaluation, using the Edinburgh Postnatal Depression Scale (EPDS). For the purpose of the analysis, mothers were considered as having symptoms of PPD if their EPDS score was ≥10 or if their score in question number 10 (self-harm) was other than 0 [17].

To test whether differences between score averages were significant, we used the Mann-Whitney *U* test [18].

### Developmental Trajectory Vectors

We described the developmental trajectory of a child by the series of its DSS values at each age step from birth to 36 months of age. Each age step *s* has an associated set of milestones *T(s)*. We further partitioned the milestones by their developmental domains, denoting by *T(s, d)* the subset of *T(s)* from domain *d* (where *d* can be either “language-social” or “motor”—an aggregation of fine-motor and gross-motor milestones). This allowed us to describe the trajectory per domain as the Developmental Trajectory Vectors (DTVs):

DTV(d) = DSS(T(s1,d),…, DSS(T(s7d)

where *s_i_* goes over the steps of 1-3 months, 3-6 months, 6-9 months, 9-12 months, 12-18 months, 18-24 months, and 24-36 months.

This representation yielded DTVs of length 7 for each child that was assessed at all age steps. For this analysis, we excluded children whose data was missing for 1 or more age steps, analyzing the remaining groups of 294,624 and 294,066 children in the motor and language-social domains, respectively.

### DTVs Clustering

We used the k-means clustering [19] to identify distinct patterns of DTVs. In addition, for sensitivity analysis of the clustering method (see Multimedia Appendix 1), we examined an alternative clustering method using a Gaussian Mixture Model [20]. Cluster validity was assessed using the Calinski-Harabasz score [21] (see Multimedia Appendix 1).

The clustering was done using only 6 of the 7 DTV entries. This is because for each domain, there is one step that included only a single milestone (for motor milestones, the 12-18 months step; for language-social milestones, the 6-9 months step), which may reduce the reliability of the results. Nonetheless, when computing cluster centroids, all entries were taken into account.

Analyses were done using Python (version 3.6.7; Python Software Foundation) with the *scikit-learn* package (version 0.23.2).

### Ethics Approval

The study protocol was approved by the Soroka University Medical Center institutional ethical committee (MHC-0014-19) and was conducted in accordance with the principles of the Declaration of Helsinki. The need for informed consent was waived owing to the use of deidentified data.

## Results

### DSS of Different Population Subgroups

Table 1 shows the main characteristics of the children in the study cohort, grouped by their age at the time of the visit at the MCHC. It was evident that the number of children who visit the MCHC decreased with the child’s age (880,688/1,052,905, 83.6% of the cohort visited at 0-12 months of age, whereas only 635,009/1,052,905, 60.3% visited at 24-36 months of age).

**Table 1:** Number of children with recorded developmental surveillance from the Israeli Ministry of Health, between July 2014 and September 2021, according to age group and stratified by child and mother characteristics. Some categories do not sum up to the total number due to missing values. Children for which the value of some characteristic is missing are not counted toward the tallies of that characteristic. Preterm children were not included in analysis, except for the analysis on gestational age.

Characteristic		Children aged 0-12 months (n=880,688), n (%)	Children aged 12-24 months (n=805,231), n (%)	Children aged 24-36 months (n=635,009), n (%)
**Developmental tracking**				
	Tracked	8595 (1)	13,711 (1.7)	13,508 (2.1)
	Not tracked	842,307 (95.6)	765,075 (95)	597,559 (94.1)
**Sex**				
	Female	429,695 (48.8)	393,115 (48.8)	309,995 (48.8)
	Male	450,993 (51.2)	412,116 (51.2)	325,014 (51.2)
**Postpartum depression**				
	Positive	27,791 (3.2)	23,719 (2.9)	17,520 (2.8)
	Negative	685,335 (77.8)	567,633 (70.5)	399,514 (62.9)
**Mother’s age (years)**				
	18-39	739,073 (83.9)	686,705 (85.3)	549,114 (86.5)
	40-50	130,270 (14.8)	110,398 (13.7)	81,249 (12.8)
**Birth weight (kg)**				
	1-2.5	30,036 (3.4)	27,761 (3.4)	22,298 (3.5)
	2.5-3	193,291 (21.9)	178,017 (22.1)	142,000 (22.4)
	3-3.5	393,690 (44.7)	360,481 (44.8)	284,226 (44.8)
	3.5-4	226,507 (25.7)	205,010 (25.5)	159,857 (25.2)
	4-4.5	44,687 (5.1)	40,366 (5)	31,412 (4.9)
	4.5-6	3929 (0.4)	3580 (0.4)	2778 (0.4)
**Child number**				
	1	316,738 (36)	310,973 (38.6)	274,206 (43.2)
	2	289,212 (32.8)	274,439 (34.1)	221,029 (34.8)
	3	167,647 (19)	142,394 (17.7)	97,590 (15.4)
**Mothers’ education**				
	Academic	267,199 (30.3)	246,190 (30.6)	198,099 (31.2)
	Tertiary education	91,060 (10.3)	79,979 (9.9)	59,102 (9.3)
	High school	233,613 (26.5)	217,952 (27.1)	176,831 (27.8)
	Elementary	18,651 (2.1)	17,273 (2.1)	13,974 (2.2)
**Gestational age (weeks; total includes preterm children: aged 0-12 months, n=943,354; aged 12-24 months, n=864,421; and aged 24-36 months, n=682,999)**				
	23-27	1410 (0.1)	1387 (0.2)	1134 (0.2)
	28-31	5135 (0.5)	4912 (0.6)	4090 (0.6)
	32-36	56,934 (6)	53,634 (6.2)	43,346 (6.3)
	37-38	228,343 (24.2)	209,255 (24.2)	166,043 (24.3)
	39-42	651,532 (69)	595,233 (68.8)	468,386 (68.5)

**Figure 2:**
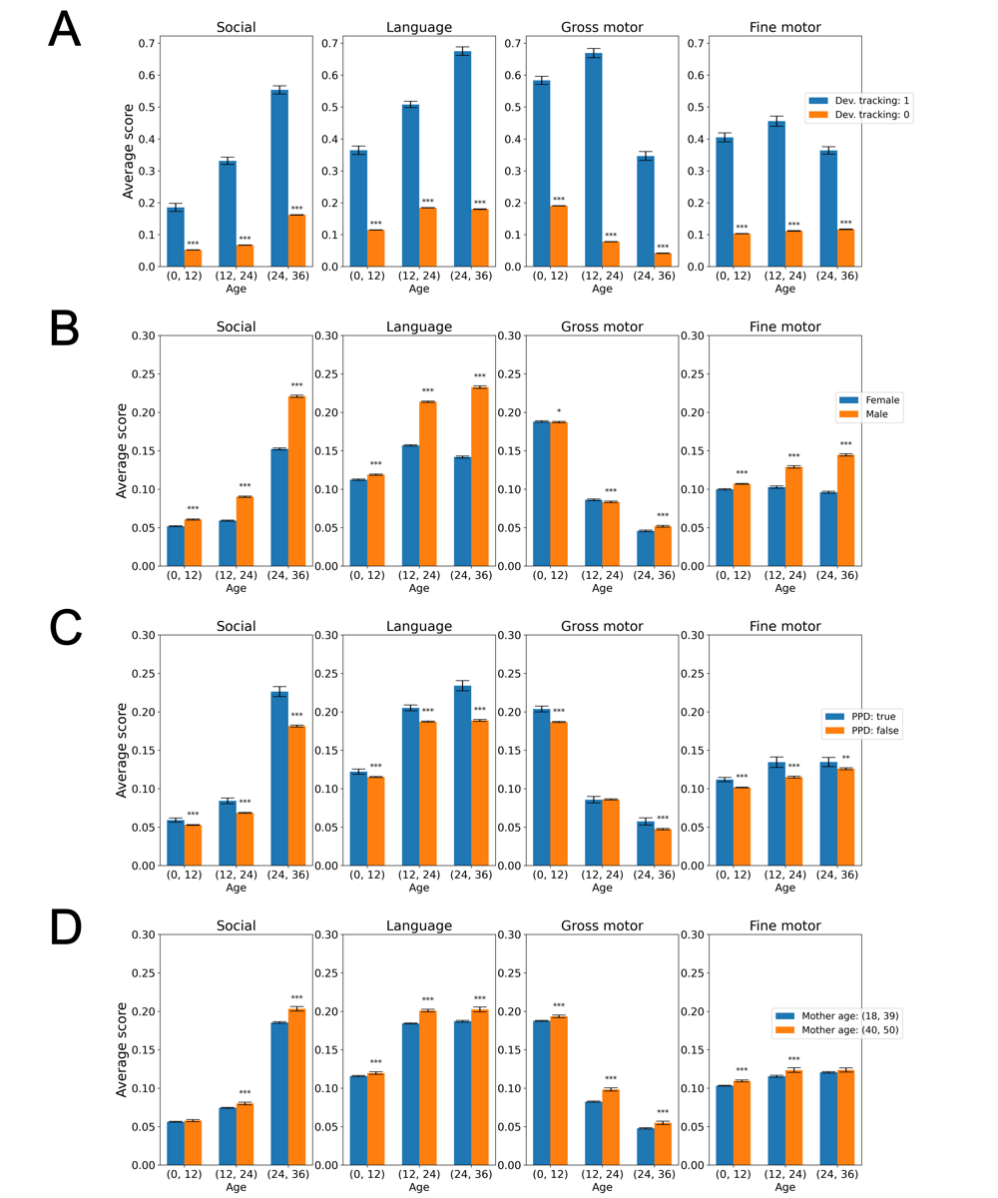
Developmental surveillance score (DSS) of binary variables. (A) Children under developmental (dev.) tracking compared to those who are not; (B) female children compared to male children; (C) children whose mothers reported postpartum depression (PPD) symptoms compared to those who did not; and (D) children of younger mothers (aged 18-39 years) compared to older mothers (aged 40-50 years). Asterisks denote a statistically significant difference between a pair of bars (*P<.05; **P<.01; ***P<.001). Based on developmental surveillance data from the Israeli Ministry of Health, between July 2014 and September 2021.

To assess the relations between the DSS and characteristics of the children or their mothers, we compared, for each domain, the average DSS of several subgroups during the first, second, and third years of life. Figure 2 shows that the average DSS was higher (worse) for children that were under designated developmental tracking, compared to the complementary group (Figure 2A). Higher DSS was evident in the following subgroups: male children (Figure 2B), children whose mothers reported symptoms of PPD (Figure 2C), and children of older mothers (Figure 2D).

**Figure 3:**
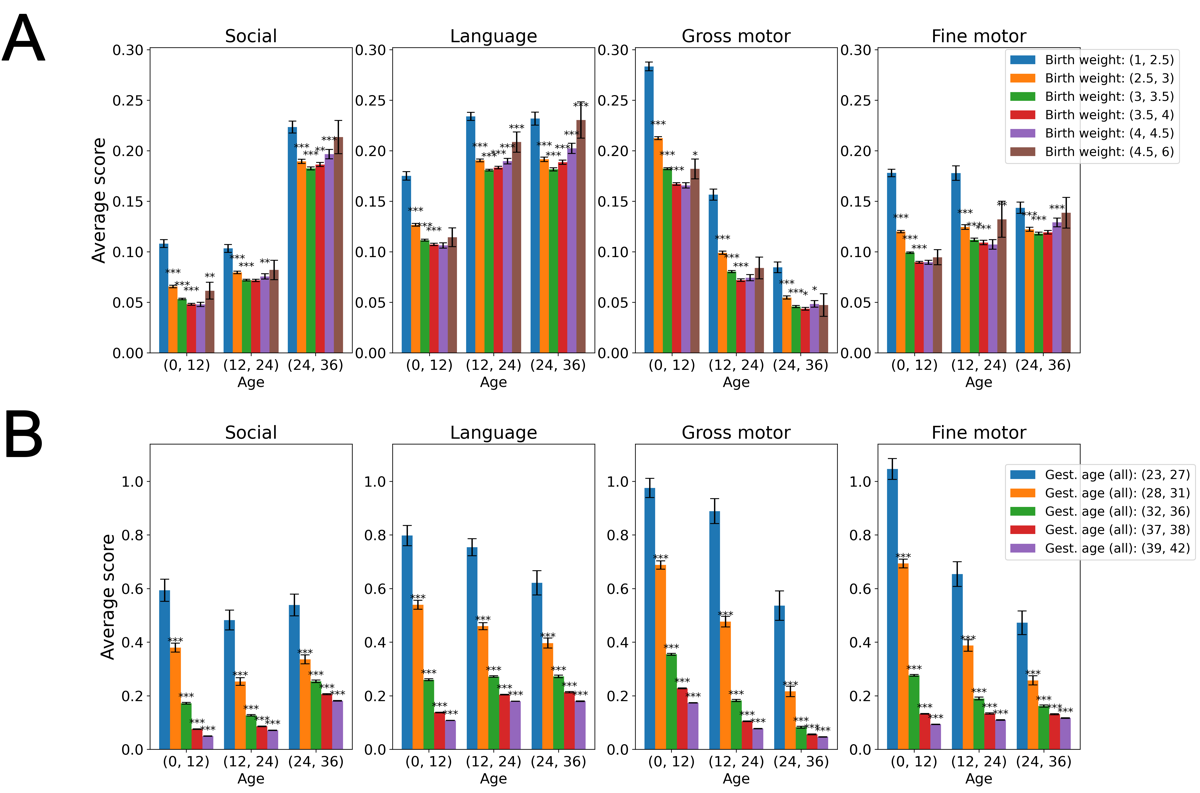
Relation between Developmental Surveillance Score (DSS) and numeric birth variables. (A) The child’s birth weight and (B) gestational (gest.) age at birth. Asterisks denote a statistically significant difference between a pair of consecutive bars (*P<.05; **P<.01; ***P<.001). Based on developmental surveillance data from the Israeli Ministry of Health, between July 2014 and September 2021.

Figure 3A demonstrates the relation between the DSS and birth weight: children with birth weight of <2.5 kg or >4.5 kg had higher average DSS than children with normative birth weight (2.5-4.5 kg). Figure 3B shows that the DSS was negatively correlated to the gestational age at birth (eg, in the first year of life, Pearson *r*=–0.2 for gross motor milestones, –0.25 for fine motor milestones, and –0.18 for language-social milestones; *P*<.001). There were marked differences between preterm and on-term children, as well as between subgroups of extremely preterm, very preterm, moderate preterm, early term, and full-term children.

**Figure 4:**
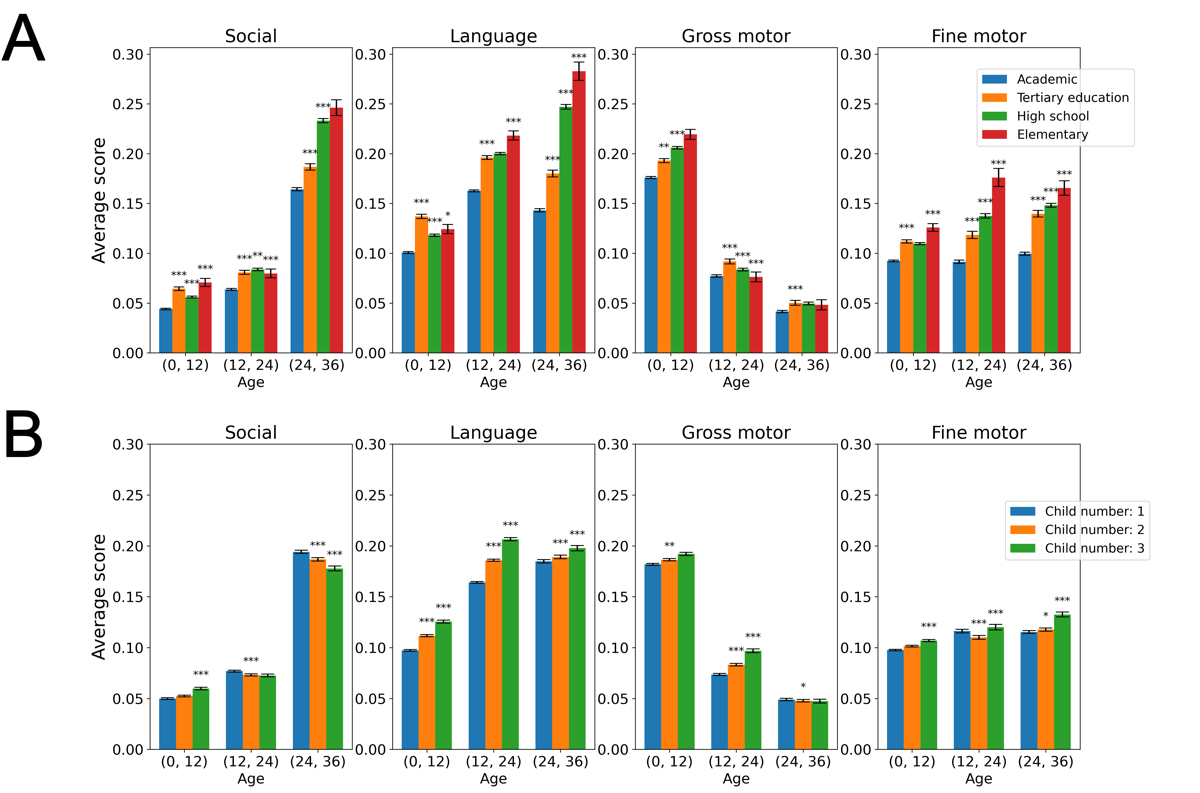
Relation between Developmental Surveillance Score (DSS) and categorical variables. (A) Maternal education level and (B) child’s birth order. Asterisks denote a statistically significant difference between a pair of consecutive bars (*P<.05; **P<.01; ***P<.001). Based on developmental surveillance data from the Israeli Ministry of Health, between July 2014 and September 2021.

Figure 4A shows the association between the DSS and the mothers’ level of education. The DSS tended to be higher among mothers with less formal education. In addition, the score appeared to be positively correlated with the child’s birth order during the first year of life (Figure 4B; Pearson *r*=0.02 for gross motor milestones, 0.03 for fine motor milestones, and 0.08 for language-social milestones; *P*<.001), with firstborn children having the least average score. This trend was maintained for the gross motor and language-social scores during the second year of life (Pearson *r*=0.03 for gross motor milestones, 0.01 for fine motor milestones, and 0.07 for language-social milestones; *P*<.001). Conversely, this correlation was evident during the third year of life only for fine-motor tasks (*r*=0.02; *P*<.001). Importantly, these correlations should be considered as affirmation for the trends suggested by the graphs—their relatively low values on these large cohorts certainly do not imply that the DSS “explains” in any way the measured characteristics.

Note that all these graphs depict average values. For the most part, children attained the assessed milestones and received a score of 0. See Table S1 in Multimedia Appendix 1 for the median and IQR values of the DSS and Figures S1-S3 in Multimedia Appendix 1 for the same analysis using DMAS instead of LMAS.

### Using the DSS to Describe Children’s Developmental Trajectories

**Figure 5:**
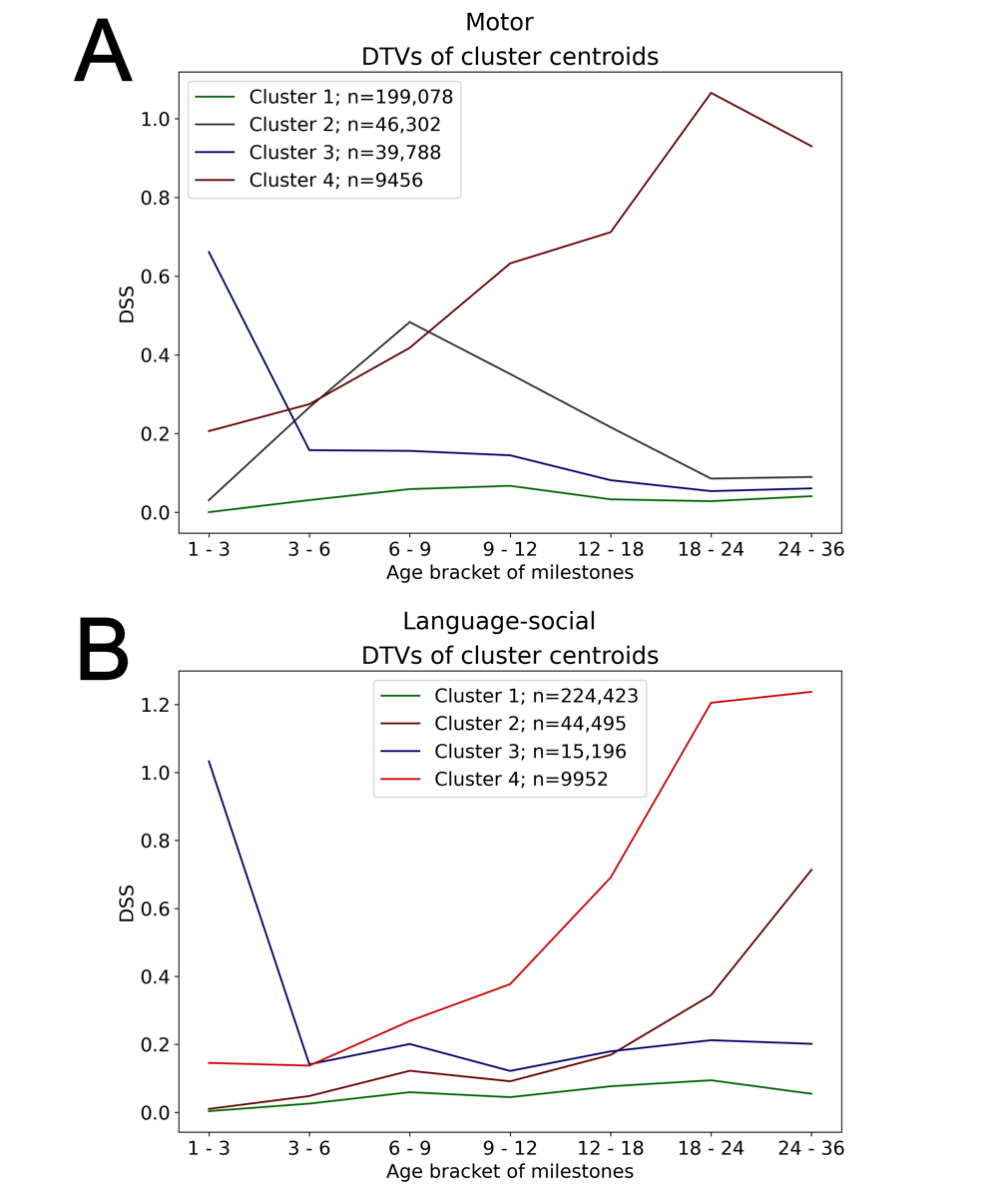
Centroids of motor Developmental Trajectory Vectors (DTV) clusters. (A) Scores derived from fine motor and gross motor milestones and (B) scores derived from language and social milestones. Centroids of clusters are (A): 1. [0.01, 0.03, 0.06, 0.07, 0.03, 0.03, 0.04]; 2. [0.03, 0.27, 0.48, 0.35, 0.22, 0.08, 0.09]; 3. [0.66, 0.16, 0.16, 0.14, 0.05, 0.05, 0.06]; 4. [0.21, 0.27, 0.42, 0.63, 0.71, 1.07, 0.93]; and (B): 1. [0, 0.03, 0.04, 0.04, 0.08, 0.09, 0.05]; 2. [0.01, 0.05, 0.11, 0.09, 0.17, 0.35, 0.71]; 3. [1.03, 0.14, 0.15, 0.12, 0.18, 0.21, 0.20]; 4. [0.15, 0.14, 0.26, 0.38, 0.69, 1.20, 1.24]. Based on developmental surveillance data from the Israeli Ministry of Health, between July 2014 and September 2021. DSS: Developmental Surveillance Score.

Figure 5 depicts the centroids derived from clustering of all children’s DTVs into 4 clusters. Both motor DTVs and language-social DTVs exhibited similar patterns. There was a single cluster of children with near-zero DSS at all age steps. This cluster included the majority of children (“adequate”; motor DTVs: 199,078/294,624, 67.6%; language-social DTVs: 224,423/294,066, 76.3%). There was a single cluster of children who were “catching up”—their DSS was initially high but tended to decrease over time. There were clusters of “worsening” children whose scores tended to increase over time (2 clusters for language-social milestones and 1 for motor milestones). For motor milestones, there was also a cluster of children whose DSS increased at an early age but then decreased back to normal values and, so, did not conform to any of these 3 patterns.

Tables 2 and 3 show the distributions of child and mother’s characteristics among the 4 different clusters in each domain. In the clusters depicting an increasing trajectory, there was an overrepresentation of male children relative to the “adequate” cluster. Specifically, in the motor domain, 50.7% (100,901/199,078) of the children in the “adequate” cluster were male, compared to 57.8% (5466/9456) in the “worsening” cluster. In the language-social domain, male children were 48.1% (107,970/224,423) of those in the “adequate” cluster, compared to 71.3% (7093/9952) and 62% (27,566/44,495) in the rapidly “worsening” and moderately “worsening” clusters, respectively. In addition, the “worsening” clusters had larger proportions of children that were born by cesarean section, had low birth weight, or were under developmental tracking.

**Table 2:** Distribution of child and mother characteristics in motor milestones clusters. Based on developmental surveillance data (N=294,624) from the Israeli Ministry of Health, between July 2014 and September 2021.

Characteristic			Cluster 1 (“adequate”; n=199,078, 67.6%), n (%)		Cluster 2 (n=46,302, 15.7%)		Cluster 3 (“catching up”; n=39,788, 13.5%), n (%)		Cluster 4 (“worsening”; n=9456, 3.2%), n (%)
**Developmental tracking**									
	Tracked	4310 (2.2)		3200 (6.9)		1853 (4.7)		2471 (26.1)	
	Not tracked	194,768 (97.8)		43,102 (93.1)		37,935 (95.3)		6985 (73.9)	
**Sex**									
	Female	98,177 (49.3)		22,783 (49.2)		19,108 (48)		3990 (42.2)	
	Male	100,901 (50.7)		23,519 (50.8)		20,680 (52)		5466 (57.8)	
**Postpartum depression**									
	Positive	7638 (3.8)		1943 (4.2)		1647 (4.1)		436 (4.6)	
	Negative	171,034 (85.9)		38,357 (82.8)		33,626 (84.5)		7887 (83.4)	
**Mother’s age (years)**									
	18-39	170,833 (85.8)		39,633 (85.6)		33,539 (84.3)		7837 (82.9)	
	40-50	26,985 (13.6)		6483 (14)		6065 (15.2)		1561 (16.5)	
**Birth weight (kg)**									
	1-2.5	6826 (3.4)		2977 (6.4)		2252 (5.7)		816 (8.6)	
	2.5-3	43,905 (22.1)		12,114 (26.2)		10,301 (25.9)		2637 (27.9)	
	3-3.5	89,570 (45)		19,910 (43)		17,283 (43.4)		3797 (40.2)	
	3.5-4	50,903 (25.6)		9864 (21.3)		8625 (21.7)		1912 (20.2)	
	4-4.5	9610 (4.8)		1845 (4)		1659 (4.2)		375 (4)	
	4.5-6	790 (0.4)		173 (0.4)		139 (0.3)		43 (0.5)	
**Child number**									
	1	88,383 (44.4)		19,530 (42.2)		17,986 (45.2)		4003 (42.3)	
	2	70,331 (35.3)		16,363 (35.3)		13,379 (33.6)		3184 (33.7)	
	3	30,518 (15.3)		7640 (16.5)		6129 (15.4)		1601 (16.9)	
**Mother’s education**									
	Academic	72,371 (36.4)		15,419 (33.3)		13,242 (33.3)		2659 (28.1)	
	Tertiary education	15,538 (7.8)		3995 (8.6)		3213 (8.1)		807 (8.5)	
	High school	57,749 (29)		14,980 (32.4)		12,127 (30.5)		3206 (33.9)	
	Elementary	4124 (2.1)		1194 (2.6)		1023 (2.6)		239 (2.5)	

**Table 3:** Distribution of child and mother characteristics in language-social milestones clusters. Based on developmental surveillance data (N=294,066) from the Israeli Ministry of Health, between July 2014 and September 2021.

Characteristic			Cluster 1 (“adequate”; n=224,423, 76.3%), n (%)		Cluster 2 (“worsening”; n=44,495, 15.1%), n (%)		Cluster 3 (“catching up”; n=15,196, 5.2%), n (%)		Cluster 4 (“worsening”; n=9952, 3.4%), n (%)
**Developmental tracking**									
	Tracked	5225 (2.3)		3349 (7.5)		678 (4.5)		2536 (25.5)	
	Not tracked	219,198 (97.7)		41,146 (92.5)		14,518 (95.5)		7416 (74.5)	
**Sex**									
	Female	116,453 (51.9)		16,929 (38)		7540 (49.6)		2859 (28.7)	
	Male	107,970 (48.1)		27,566 (62)		7656 (50.4)		7093 (71.3)	
**Postpartum depression**									
	Positive	8368 (3.7)		2048 (4.6)		688 (4.5)		563 (5.7)	
	Negative	191,397 (85.3)		37,869 (85.1)		12,814 (84.3)		8341 (83.8)	
**Mother’s age (years)**									
	18-39	192,242 (85.7)		37,812 (85)		13,115 (86.3)		8199 (82.4)	
	40-50	30,837 (13.7)		6473 (14.5)		2011 (13.2)		1690 (17)	
**Birth weight (kg)**									
	1-2.5	8866 (4)		1922 (4.3)		1382 (9.1)		690 (6.9)	
	2.5-3	51,919 (23.1)		10,117 (22.7)		4345 (28.6)		2483 (24.9)	
	3-3.5	100,692 (44.9)		19,246 (43.3)		6252 (41.1)		4109 (41.3)	
	3.5-4	54,809 (24.4)		11,185 (25.1)		2854 (18.8)		2258 (22.7)	
	4-4.5	10,176 (4.5)		2295 (5.2)		497 (3.3)		489 (4.9)	
	4.5-6	828 (0.4)		219 (0.5)		48 (0.3)		50 (0.5)	
**Child number**									
	1	101,311 (45.1)		18,400 (41.4)		5972 (39.3)		4245 (42.7)	
	2	78,671 (35.1)		16,053 (36.1)		5059 (33.3)		3318 (33.3)	
	3	33,612 (15)		7590 (17.1)		2735 (18)		1695 (17)	
**Mother’s education**									
	Academic	84,575 (37.7)		12,518 (28.1)		4378 (28.8)		2062 (20.7)	
	Tertiary education	17,411 (7.8)		3788 (8.5)		1411 (9.3)		849 (8.5)	
	High school	62,599 (27.9)		16,805 (37.8)		4574 (30.1)		4092 (41.1)	
	Elementary	4426 (2)		1359 (3.1)		450 (3)		371 (3.7)	

In Multimedia Appendix 1, we demonstrate that qualitatively, these results were consistent over different range of clusters number, as well as when using an alternative clustering method.

## Discussion

The goal of this study was to construct a DSS that can be used for comparative tracking of children’s development, quantifying milestones attainment in a concise and straightforward way. We presented a simple methodology for calculating the DSS, a quantitative developmental surveillance score that aggregates age-dependent milestones results over a chosen time frame and domain into a single score. To demonstrate its coherence, we explored 2 main use cases for this score: comparing its value among subpopulations and using it to depict the developmental trajectory of individuals. We demonstrated that the DSS reflects known associations between developmental status and characteristics of the child and mother and its potential for suggesting possible new associations and insights, which may be a stepping stone for further research.

Children who have been referred to developmental tracking, indicating that they are likely to exhibit a developmental delay, had on average a much higher score than their counterparts, at all 3 examined age groups and for all 3 developmental domains. In addition, the score was shown to reflect previously reported associations between developmental status and the child’s sex [22-28], birth weight [27,29-32], gestational age [30-34], birth order [32,35-37], maternal age [31,38,39], maternal education [28,31,32,38], and maternal symptoms of PPD [28,40-43].

For some of these variables, the DSS suggests a possible association with developmental delays, depicting different score distributions among subgroups stratified by the variable, even within the normal range. For example, it is well established that low birth weight is associated with developmental delays [27,29-32], yet the results herein suggest that this may also be true for birth weight within the lower normal range (2.5-3 kg) and for birth weight above the normal range (more than 4.5 kg). Similarly, although the scores of preterm children are higher than full-term children, there is a gradual decrease in the average score by the level of prematurity (extreme preterm, very preterm, and late preterm children), as well as a difference between early term and full-term children.

At the same time, some characteristics show a more complex behavior; for example, the DSS tends to be positively correlated with the child’s order, yet for language-social tasks evaluated at 24-36 months of age, the correlation becomes negative. Indeed, although previous work generally associate primipara with lower risk for development delay [32,35,36], Oshima-Takane et al [37], who focused on language development at 21 and 24 months of age, observed higher language skills among second-born children.

Cluster analysis consistently identified 3 types of developmental trajectories: 1 cluster of children who succeed in attaining nearly all milestones, containing most of the children; 1 cluster of children who tend to fail early-age milestones but show improvement over time and succeed in attaining later milestones; and 1 or more clusters of children whose performance grow worse over time, with different clusters depicting different severities of failures. These clusters correspond to common types of developmental patterns observed in clinical practice; although, importantly, not all clusters can be categorized as 1 of these 3 types. Future work may use these clusters as class labels, in an attempt to predict the developmental trajectory type of a child at an early age and, accordingly, consider timely intervention when needed.

This work has several limitations. Importantly, the main goal was to present the DSS and show that it is consistent with current knowledge on risk factors for developmental delay such as low birth weight, preterm birth, older maternal age, symptoms of PPD, or lower level of maternal education, as well as to suggest interesting new observations. It is not proposed as a screening tool, and although we demonstrated its rationale and coherence, we lacked a “ground truth” of developmental delay for validating the score against. Future work should aim to assess the score’s potential contribution to the clinical workflow of developmental assessment, for example, by comparing it to developmental screening tools such as the Bayley [13] and Denver [8,11] scales, as well as to developmental outcomes beyond those in the current data set, such as a diagnosis of autism*.*

Such a comparison is also needed for the calibration of the method with respect to milestones and age windows used to derive the score. For example, deriving the score by averaging milestone attainment during a full year implicitly assumes that a single number can represent the developmental delay over this entire period. Conversely, calculating a new score per visit does not take into account valuable information from past evaluations.

Another limitation stems from the use of slightly different cohorts for each age group. As depicted in Table 1, the cohorts differ in size and some of the characteristics, which may introduce some bias to the comparisons of age groups. However, as most of the presented results compare stratified population groups, the existence of similar differences in each age group strengthens the derived observations.

The results described herein pertain to the milestones used in Israeli MCHCs and the age thresholds computed in the THIS developmental scale [9]. Generalizing these results to other settings can be done by adopting the same methodology but would require having, or constructing, a developmental scale that is suitable for that setting. With such a scale at hand, one can compute a DSS from milestone attainment data by comparing them to the age thresholds and defining the score accordingly.

Taken together, our results suggest the potential usefulness of incorporating the DSS into the developmental surveillance workflow. We envision it as being computed automatically once a child’s electronic health record is updated with new milestone attainment results and compared to the child’s trajectory of past achievements, as well as to the population’s norm. In cases where the score deviates significantly on either count, the system would notify the nurse, possibly leading to a more thorough evaluation. When calibrated correctly, such a system could identify developmental delays in a timely manner and foster interventions for improving the prospective outcomes.

## Appendix

Multimedia Appendix 1 Supplemental materials and results.

## Abbreviations

ASQ-3: Ages & Stages Questionnaires
DMAS: Discrete Milestone Attainment Score
DSS: Developmental Surveillance Score
DTV: Developmental Trajectory Vector
EPDS: Edinburgh Postnatal Depression Scale
LMAS: Linearized Milestone Attainment Score
MCHC: maternal child health clinic
PPD: postpartum depression
THIS: Tipat Halav Israel Surveillance

## Data Availability

The deidentified patient-level data used for this study contain sensitive information and therefore are not available outside the secured research environment of the Israeli Ministry of Health. Summary aggregate-level data and analysis code for this study can be made available upon reasonable request to the corresponding author.

## References

[1] Guevara JP, Gerdes M, Localio R, Huang YV, Pinto-Martin J, Minkovitz CS, Hsu D, Kyriakou L, Baglivo S, Kavanagh J, Pati S (2013). Effectiveness of developmental screening in an urban setting. Pediatrics.

[2] Lipkin PH, Macias MM, Council on Children With Disabilities, Section on Developmental and Behavioral Pediatrics (2020). Promoting optimal development: identifying infants and young children with developmental disorders through developmental surveillance and screening. Pediatrics.

[3] Hirai AH, Kogan MD, Kandasamy V, Reuland C, Bethell C (2018). Prevalence and variation of developmental screening and surveillance in early childhood. JAMA Pediatrics.

[4] Barger B, Rice C, Wolf R, Roach A (2018). Better together: developmental screening and monitoring best identify children who need early intervention. Disability Health Journal.

[5] Reichow B, Hume K, Barton EE, Boyd BA (2018). Early intensive behavioral intervention (EIBI) for young children with autism spectrum disorders (ASD). Cochrane Database Syst Rev.

[6] Council on Children With Disabilities, Section on Developmental Behavioral Pediatrics, Bright Futures Steering Committee, Medical Home Initiatives for Children With Special Needs Project Advisory Committee (2006). Identifying infants and young children with developmental disorders in the medical home: an algorithm for developmental surveillance and screening. Pediatrics.

[7] Hagan JF, Shaw JS, Duncan RM (2017). Bright futures: guidelines for health supervision of infants, children, and adolescents. Vanderbilt University.

[8] Frankenburg WK, Dodds J, Archer P, Shapiro H, Bresnick B (1992). The Denver II: a major revision and restandardization of the Denver Developmental Screening Test. Pediatrics.

[9] Sudry T, Zimmerman DR, Yardeni H, Joseph A, Baruch R, Grotto I, Greenberg D, Eilenberg R, Amit G, Akiva P, Tsadok MA, Rize Y, Zaworbach H, Uziel M, Ben Moshe D, Lior Sadaka I, Bachmat E, Freedman J, Sadaka Y (2022). Standardization of a Developmental Milestone Scale using data from children in Israel. JAMA Network Open.

[10] Sheldrick RC, Perrin EC (2013). Evidence-based milestones for surveillance of cognitive, language, and motor development. Acad Pediatr.

[11] Frankenburg WK, Dodds JB (1967). The Denver Developmental Screening Test. J Pediatr.

[12] Squires JK, Bricker D (2009). Ages & Stages Questionnaires. Questionnaires Set. 3rd ed.

[13] Aylward GP, Zhu J (2019). The Bayley Scales: clarification for clinicians and researchers. Pearson Assessments.

[14] Weber AM, Rubio-Codina M, Walker SP, van Buuren S, Eekhout I, Grantham-McGregor SM, Araujo MC, Chang SM, Fernald LC, Hamadani JD, Hanlon C, Karam SM, Lozoff B, Ratsifandrihamanana L, Richter L, Black MM, Working group membersdata contributors (2019). The D-score: a metric for interpreting the early development of infants and toddlers across global settings. BMJ Glob Health.

[15] (2022). The Israeli development scale for ages 0-5 years. KI Institute.

[16] Quinn JA, Munoz FM, Gonik B, Frau L, Cutland C, Mallett-Moore T, Kissou A, Wittke F, Das M, Nunes T, Pye S, Watson W, Ramos AA, Cordero JF, Huang W, Kochhar S, Buttery J, Brighton Collaboration Preterm Birth Working Group (2016). Vaccine.

[17] Murray L, Carothers AD (1990). The validation of the Edinburgh Post-natal Depression Scale on a community sample. Br J Psychiatry.

[18] Mann HB, Whitney DR (1947). On a test of whether one of two random variables is stochastically larger than the other. Ann Math Statist.

[19] Lloyd S (1982). Least squares quantization in PCM. IEEE Trans Inform Theory.

[20] Bishop CM (2016). attern Recognition and Machine Learning. Softcover reprint of the original 1st edition 2006 (corrected at 8th printing 2009).

[21] Calinski T, Harabasz J (1974). A dendrite method for cluster analysis. Comm in Stats - Simulation & Comp.

[22] Hyde JS, Linn MC (1988). Gender differences in verbal ability: a meta-analysis. Psychol Bull.

[23] Rinaldi P, Pasqualetti P, Volterra V, Caselli MC (2023). Gender differences in early stages of language development. some evidence and possible explanations. J Neurosci Res.

[24] Dinkel D, Snyder K (2020). Exploring gender differences in infant motor development related to parent's promotion of play. Infant Behav Dev.

[25] Escolano-Pérez Elena, Sánchez-López CR, Herrero-Nivela ML (2021). Early environmental and biological influences on preschool motor skills: implications for early childhood care and education. Front Psychol.

[26] WHO Multicentre Growth Reference Study Group (2006). Assessment of sex differences and heterogeneity in motor milestone attainment among populations in the WHO Multicentre Growth Reference Study. Acta Paediatr Suppl.

[27] To T, Guttmann A, Dick PT, Rosenfield JD, Parkin PC, Cao H, Vydykhan TN, Tassoudji M, Harris JK (2004). What factors are associated with poor developmental attainment in young Canadian children?. Can J Public Health.

[28] To T, Guttmann A, Dick PT, Rosenfield JD, Parkin PC, Tassoudji M, Vydykhan TN, Cao H, Harris JK (2004). Risk markers for poor developmental attainment in young children: results from a longitudinal national survey. Arch Pediatr Adolesc Med.

[29] Lima MC, Eickmann SH, Lima ACV, Guerra MQ, Lira PIC, Huttly SRA, Ashworth A (2004). Determinants of mental and motor development at 12 months in a low income population: a cohort study in northeast Brazil. Acta Paediatr.

[30] Drozd-Dąbrowska M, Trusewicz R, Ganczak M (2018). Selected risk factors of developmental delay in Polish infants: a case-control study. Int J Environ Res Public Health.

[31] Ozkan M, Senel S, Arslan EA, Karacan CD (2012). The socioeconomic and biological risk factors for developmental delay in early childhood. Eur J Pediatr.

[32] Hediger ML, Overpeck MD, Ruan WJ, Troendle JF (2002). Birthweight and gestational age effects on motor and social development. Paediatr Perinat Epidemiol.

[33] Hochstedler KA, Bell G, Park H, Ghassabian A, Bell EM, Sundaram R, Grantz KL, Yeung EH (2021). Gestational age at birth and risk of developmental delay: the Upstate KIDS study. Am J Perinatol.

[34] Schonhaut L, Armijo I, Pérez M (2015). Gestational age and developmental risk in moderately and late preterm and early term infants. Pediatrics.

[35] Gayraud F, Kern S (2007). Influence of preterm birth on early lexical and grammatical acquisition. First Language.

[36] Alvares GA, Licari MK, Stevenson PG, Bebbington K, Cooper MN, Glasson EJ, Tan DW, Uljarević M, Varcin KJ, Wray J, Whitehouse AJO (2021). Investigating associations between birth order and autism diagnostic phenotypes. J Child Psychol Psychiatry.

[37] Oshima-Takane Y, Goodz E, Derevensky JL (1996). Birth order effects on early language development: do secondborn children learn from overheard speech?. Child Development.

[38] Demirci A, Kartal M (2018). Sociocultural risk factors for developmental delay in children aged 3-60 months: a nested case-control study. Eur J Pediatr.

[39] Geronimus AT, Korenman S, Hillemeier MM (1994). Does young maternal age adversely affect child development? evidence from cousin comparisons in the United States. Popul Dev Rev.

[40] Murray L, Cooper PJ (1997). Effects of postnatal depression on infant development. Arch Dis Child.

[41] Lubotzky-Gete S, Ornoy A, Grotto I, Calderon-Margalit R (2021). Postpartum depression and infant development up to 24 months: a nationwide population-based study. J Affect Disord.

[42] Grace SL, Evindar A, Stewart DE (2003). The effect of postpartum depression on child cognitive development and behavior: a review and critical analysis of the literature. Arch Womens Ment Health.

[43] Deave T, Heron J, Evans J, Emond A (2008). The impact of maternal depression in pregnancy on early child development. BJOG.

