# Supplementary material for: A Developmental Surveillance Score for Quantitative Monitoring of Early Childhood Milestone Attainment: Algorithm Development and Validation
Source: JMIR Public Health Surveill. 2023 Aug 18;9:e47315. doi: 10.2196/47315 (PMC10474508; doi:10.2196/47315)
Supplement: Multimedia Appendix 1 [file publichealth_v9i1e47315_app1.docx]

# Supplementary materials and results

## Median and IQRs of DSS

Stratification of the data according to various characteristics of the child and mother yields average DSS scores that are consistent with known associations between these characteristics and developmental delay. However, as typically children succeed in attaining milestones, Table S1 summarizes the median values and IQRs associated with the graphs in Figures 2-4. In most cases the 25% quantile and the median are 0, and for motor tasks at ages 12-36 months even the 75% quantile is usually 0. In other words, even in subsets of the population which are at an elevated risk for developmental delay, most children attain the milestone and get a score of 0.

## Computing DSS: an Example

Consider Alice, a child who visits the MCHC at age 9.5 months, and then again at age 11 month. In the first visit she is assessed on the following milestones: “Responds differently to familiar and stranger” (language-social), “Feeds self” (fine motor), “Uses thumb-fingers grasp” (fine motor), “Understands simple instructions” (language-social) and “Gets to sit without support” (Gross motor). Alice achieves the first two milestones, and fails the latter three. On her subsequent visit, she is assessed on the milestones which she failed, which she attains, and also on the milestone of “Says one word or pronounces meaningful sounds” (language-social), which she fails to attain.

To compute her score in the language-social domain, we consider the relevant tasks - “Responds differently to familiar and stranger”, “Understands simple instructions” (which was assessed twice) and “Says one word or pronounces meaningful sounds”. In total there are four assessments. In two, the milestone was attained, giving a DMAS of zero. According to the THIS scale, the milestone “Understands simple instructions” is attained by 75%-90% of children aged 9 months, so Alice’s failure defines a DMAS of 2. The milestone “Says one word or pronounces meaningful sounds” is attained by 90%-95% of children aged 11 months, so Alice’s failure here defines a DMAS of 3. Her DSS for language-social development is there for (2+3)/4 = 1.2, where we divide by 4 since that is the total number of assessments in this domain.

In the fine-motor domain, Alice was assessed on two milestones, one which she attained (DMAS of 0), and one which she failed, whereas 90%-95% of children her age attain it - yielding a DMAS of 3. In total, her DSS for fine-motor skills is 3/2 = 1.5.

In the gross motor domain, Alice ailed the single task she was assessed on, at an age where less than 75% of children attain the milestone. This gives a DMAS of 1, which is also the DSS since there is only one task.

Computing LMAS is a little more tricky. For attained milestones, LMAS is 0, just like DMAS. As we noted, the milestone “Understands simple instructions” which Alice failed to attain at 9.5 months are attained by 75%-90% of children at this age. Why now need to consult the THIS scale to see what is the lowest age for which this is the milestone attainment rate, and what is the highest age. It turns out that this rate is only relevant for children who are at least 9 months old, but younger than 10 months. Since Alice’s age is in the middle of this range, her LMAS for this task 1.5. Her failure at the milestone “Says one word or pronounces meaningful sounds” at age 11 month is exactly at the turning point between the 75%-90% bracket and the 90%-95%, so that gives an LMAS of 2. Her language-social DSS using LMAS is therefore (1.5+2)/4 = 0.875.

Similarly, “Uses thumb-fingers grasp” was failed in the middle of the age range for the 75%-90% bracket yielding an LMAS of 1.5, and “Gets to sit without support” at the turning point between the 75%-90% bracket and the 90%-95%, yielding an LMAS of 2. Hence, her LMAS-based DSS for fine-motor skills is 1.5/2 = 0.75, and for gross motor it is 2.

|  | gross motor Age 0-12 months | gross motor Age 12-24 months | gross motor Age 24-36 months | fine motor Age 0-12 months | fine motor Age 12-24 months | fine motor Age 24-36 months | language-social Age 0-12 months | language-social Age 12-24 months | language-social Age 24-36 months |
| --- | --- | --- | --- | --- | --- | --- | --- | --- | --- |
| Dev. tracking: Tracked | 0.46 [0.00-0.91] | 0.20 [0.00-1.20] | 0.00 [0.00-0.00] | 0.00 [0.00-0.59] | 0.00 [0.00-0.50] | 0.00 [0.00-0.54] | 0.00 [0.00-0.35] | 0.25 [0.00-0.64] | 0.38 [0.00-1.08] |
| Dev. tracking: Not tracked | 0.00 [0.00-0.31] | 0.00 [0.00-0.00] | 0.00 [0.00-0.00] | 0.00 [0.00-0.12] | 0.00 [0.00-0.00] | 0.00 [0.00-0.00] | 0.00 [0.00-0.00] | 0.09 [0.00-0.21] | 0.00 [0.00-0.20] |
| Gender: Female | 0.00 [0.00-0.31] | 0.00 [0.00-0.00] | 0.00 [0.00-0.00] | 0.00 [0.00-0.12] | 0.00 [0.00-0.00] | 0.00 [0.00-0.00] | 0.00 [0.00-0.00] | 0.00 [0.00-0.19] | 0.00 [0.00-0.17] |
| Gender: Male | 0.00 [0.00-0.31] | 0.00 [0.00-0.00] | 0.00 [0.00-0.00] | 0.00 [0.00-0.12] | 0.00 [0.00-0.00] | 0.00 [0.00-0.00] | 0.00 [0.00-0.08] | 0.09 [0.00-0.25] | 0.00 [0.00-0.31] |
| PPD: Positive | 0.00 [0.00-0.33] | 0.00 [0.00-0.00] | 0.00 [0.00-0.00] | 0.00 [0.00-0.14] | 0.00 [0.00-0.00] | 0.00 [0.00-0.00] | 0.00 [0.00-0.09] | 0.09 [0.00-0.23] | 0.00 [0.00-0.31] |
| PPD: Negative | 0.00 [0.00-0.31] | 0.00 [0.00-0.00] | 0.00 [0.00-0.00] | 0.00 [0.00-0.13] | 0.00 [0.00-0.00] | 0.00 [0.00-0.00] | 0.00 [0.00-0.00] | 0.09 [0.00-0.21] | 0.00 [0.00-0.23] |
| Mother Age: (18, 39) | 0.00 [0.00-0.31] | 0.00 [0.00-0.00] | 0.00 [0.00-0.00] | 0.00 [0.00-0.12] | 0.00 [0.00-0.00] | 0.00 [0.00-0.00] | 0.00 [0.00-0.00] | 0.09 [0.00-0.21] | 0.00 [0.00-0.23] |
| Mother Age: (40, 50) | 0.00 [0.00-0.32] | 0.00 [0.00-0.00] | 0.00 [0.00-0.00] | 0.00 [0.00-0.13] | 0.00 [0.00-0.00] | 0.00 [0.00-0.00] | 0.00 [0.00-0.05] | 0.09 [0.00-0.23] | 0.00 [0.00-0.26] |
| Birth weight: (1, 2.5) | 0.16 [0.00-0.45] | 0.00 [0.00-0.00] | 0.00 [0.00-0.00] | 0.00 [0.00-0.26] | 0.00 [0.00-0.00] | 0.00 [0.00-0.00] | 0.00 [0.00-0.19] | 0.09 [0.00-0.26] | 0.00 [0.00-0.30] |
| Birth weight: (2.5, 3) | 0.00 [0.00-0.35] | 0.00 [0.00-0.00] | 0.00 [0.00-0.00] | 0.00 [0.00-0.15] | 0.00 [0.00-0.00] | 0.00 [0.00-0.00] | 0.00 [0.00-0.09] | 0.09 [0.00-0.22] | 0.00 [0.00-0.24] |
| Birth weight: (3, 3.5) | 0.00 [0.00-0.31] | 0.00 [0.00-0.00] | 0.00 [0.00-0.00] | 0.00 [0.00-0.12] | 0.00 [0.00-0.00] | 0.00 [0.00-0.00] | 0.00 [0.00-0.00] | 0.08 [0.00-0.21] | 0.00 [0.00-0.22] |
| Birth weight: (3.5, 4) | 0.00 [0.00-0.30] | 0.00 [0.00-0.00] | 0.00 [0.00-0.00] | 0.00 [0.00-0.11] | 0.00 [0.00-0.00] | 0.00 [0.00-0.00] | 0.00 [0.00-0.00] | 0.09 [0.00-0.21] | 0.00 [0.00-0.23] |
| Birth weight: (4, 4.5) | 0.00 [0.00-0.29] | 0.00 [0.00-0.00] | 0.00 [0.00-0.00] | 0.00 [0.00-0.10] | 0.00 [0.00-0.00] | 0.00 [0.00-0.00] | 0.00 [0.00-0.00] | 0.09 [0.00-0.22] | 0.00 [0.00-0.26] |
| Birth weight: (4.5, 6) | 0.00 [0.00-0.30] | 0.00 [0.00-0.00] | 0.00 [0.00-0.00] | 0.00 [0.00-0.00] | 0.00 [0.00-0.00] | 0.00 [0.00-0.00] | 0.00 [0.00-0.00] | 0.09 [0.00-0.24] | 0.00 [0.00-0.29] |
| Child number: 1 | 0.00 [0.00-0.30] | 0.00 [0.00-0.00] | 0.00 [0.00-0.00] | 0.00 [0.00-0.12] | 0.00 [0.00-0.00] | 0.00 [0.00-0.00] | 0.00 [0.00-0.00] | 0.00 [0.00-0.20] | 0.00 [0.00-0.23] |
| Child number: 2 | 0.00 [0.00-0.31] | 0.00 [0.00-0.00] | 0.00 [0.00-0.00] | 0.00 [0.00-0.12] | 0.00 [0.00-0.00] | 0.00 [0.00-0.00] | 0.00 [0.00-0.00] | 0.09 [0.00-0.21] | 0.00 [0.00-0.24] |
| Child number: 3 | 0.00 [0.00-0.32] | 0.00 [0.00-0.00] | 0.00 [0.00-0.00] | 0.00 [0.00-0.13] | 0.00 [0.00-0.00] | 0.00 [0.00-0.00] | 0.00 [0.00-0.00] | 0.09 [0.00-0.24] | 0.00 [0.00-0.23] |
| Education: Academic | 0.00 [0.00-0.30] | 0.00 [0.00-0.00] | 0.00 [0.00-0.00] | 0.00 [0.00-0.12] | 0.00 [0.00-0.00] | 0.00 [0.00-0.00] | 0.00 [0.00-0.00] | 0.07 [0.00-0.20] | 0.00 [0.00-0.19] |
| Education: Tertiary Education | 0.00 [0.00-0.32] | 0.00 [0.00-0.00] | 0.00 [0.00-0.00] | 0.00 [0.00-0.14] | 0.00 [0.00-0.00] | 0.00 [0.00-0.00] | 0.00 [0.00-0.09] | 0.09 [0.00-0.23] | 0.00 [0.00-0.23] |
| Education: High School | 0.00 [0.00-0.34] | 0.00 [0.00-0.00] | 0.00 [0.00-0.00] | 0.00 [0.00-0.13] | 0.00 [0.00-0.00] | 0.00 [0.00-0.00] | 0.00 [0.00-0.00] | 0.09 [0.00-0.23] | 0.00 [0.00-0.33] |
| Education: Elementary | 0.00 [0.00-0.35] | 0.00 [0.00-0.00] | 0.00 [0.00-0.00] | 0.00 [0.00-0.16] | 0.00 [0.00-0.00] | 0.00 [0.00-0.00] | 0.00 [0.00-0.00] | 0.09 [0.00-0.24] | 0.00 [0.00-0.38] |
| Gestational age: (23, 27) | 0.97 [0.48-1.40] | 0.72 [0.00-1.50] | 0.00 [0.00-0.79] | 1.07 [0.46-1.53] | 0.00 [0.00-1.08] | 0.00 [0.00-0.90] | 0.60 [0.00-1.16] | 0.54 [0.24-0.99] | 0.38 [0.00-0.95] |
| Gestational age: (28, 31) | 0.63 [0.25-1.02] | 0.00 [0.00-0.80] | 0.00 [0.00-0.00] | 0.66 [0.00-1.07] | 0.00 [0.00-0.51] | 0.00 [0.00-0.00] | 0.36 [0.00-0.77] | 0.29 [0.09-0.59] | 0.16 [0.00-0.59] |
| Gestational age: (32, 36) | 0.28 [0.00-0.57] | 0.00 [0.00-0.00] | 0.00 [0.00-0.00] | 0.11 [0.00-0.45] | 0.00 [0.00-0.00] | 0.00 [0.00-0.00] | 0.00 [0.00-0.38] | 0.13 [0.00-0.33] | 0.00 [0.00-0.38] |
| Gestational age: (37, 38) | 0.00 [0.00-0.37] | 0.00 [0.00-0.00] | 0.00 [0.00-0.00] | 0.00 [0.00-0.18] | 0.00 [0.00-0.00] | 0.00 [0.00-0.00] | 0.00 [0.00-0.11] | 0.09 [0.00-0.24] | 0.00 [0.00-0.27] |
| Gestational age: (39, 42) | 0.00 [0.00-0.30] | 0.00 [0.00-0.00] | 0.00 [0.00-0.00] | 0.00 [0.00-0.11] | 0.00 [0.00-0.00] | 0.00 [0.00-0.00] | 0.00 [0.00-0.00] | 0.08 [0.00-0.21] | 0.00 [0.00-0.22] |

Table S1: Median and IQR scores by age and characteristics of the child and mother, corresponding to figures 2-4.

## Consistency of cluster types

We observed that when clustering DTVs using k-means with k=4, we obtain a single, big cluster whose centroid’s DSS trajectory is near 0 at all milestones; a single cluster whose centroid has high DSS on early-age milestones which then decreases at later milestones; and one or two clusters where the centroid’s DSS tends to increase over time. We denote these types of trajectories as “adequate”, “catching up” and “worsening”, respectively, and ask whether this phenomenon is particular to k-means clustering with k=4, or consistently arises from clustering the data, regardless of the clustering details.

In order to answer this question, we first need to formally define what we mean by “adequate”, “catching up” and “worsening”. Based on examination of the centroids of the 4 clusters, we suggest this ad-hoc heuristic definition:

1. We say that a DTV is “adequate” if all its entries are at most 0.2.
2. Consider all pairs of steps (*i*,*j*) such that step i occurs at some point before step j. We say that a DTV is “worsening” if for at least 0.75 of such pairs, where the values of entries *i* and *j* are different, entry *i* is smaller than entry *j*, and in addition some entry is at least 0.5.
3. Consider all pairs of consecutive steps (*i*,*j*). We say that a DTV is “catching up” if:
   1. The first entry is higher than 0.5
   2. For most pairs where the values of entries *i* and *j* are different, entry *i* is bigger than entry *j*.
   3. The average difference between entry *i* and entry *j* is at least 0.1.
4. We say that a trajectory is “undefined” if none of the above holds.

Table S2 lists the distribution of such labels among the different clusters’ centroids, for varying values of k. We see that there is always one cluster with an “adequate” centroid, and usually one cluster labeled “catching up”. The number of “increasing” clusters is more varied – up to 4 in the case of 9 lingo-social clusters – which may reflect different severities of increasing DDSs. A similar trend is observed when using GMM clustering (Table S3), though for a low number of clusters, clusters whose centroids are “increasing” or “catching up” are not always observed.

These two tables also include the Calinski-Harabasz score, which measures the goodness of a clustering (higher is better) based on the ratio between inter-cluster distances and intra-cluster distances.

|  | Fine and gross motor | | | Language-social | | | **Calinski Harabasz Score** | |
| --- | --- | --- | --- | --- | --- | --- | --- | --- |
| Number of clusters | Adequate | worsening | Catching up | Adequate | worsening | Catching up | Fine and gross motor | Language-social |
| 3 | 1 | 1 | 1 | 1 | 1 | 1 | 59,336 | 81,710 |
| 4 | 1 | 1 | 1 | 1 | 2 | 1 | 55,355 | 71,739 |
| 5 | 1 | 2 | 1 | 1 | 2 | 1 | 50,210 | 63,830 |
| 6 | 1 | 2 | 1 | 1 | 2 | 1 | 48,532 | 59,400 |
| 7 | 1 | 2 | 1 | 1 | 3 | 1 | 47,728 | 55,576 |
| 8 | 1 | 2 | 1 | 1 | 3 | 1 | 45,712 | 51,464 |
| 9 | 1 | 3 | 1 | 1 | 4 | 1 | 43,502 | 48,522 |
| 10 | 1 | 3 | 2 | 1 | 3 | 1 | 42,865 | 45,521 |

Table S2: Number of clusters of each label, for varying number of clusters using K-means, and the Clainski-Harabasz score for each clustering

|  | Fine and gross motor | | | language-social | | | **Calinski Harabasz Score** | |
| --- | --- | --- | --- | --- | --- | --- | --- | --- |
| Number of clusters | Adequate | worsening | Catching up | Adequate | worsening | Catching up | Fine and gross motor | Language-social |
| 3 | 1 | 1 | 0 | 1 | 1 | 0 | 39,709 | 47,706 |
| 4 | 1 | 0 | 0 | 1 | 1 | 1 | 31,867 | 35,796 |
| 5 | 1 | 1 | 1 | 1 | 0 | 0 | 30,539 | 31,794 |
| 6 | 1 | 1 | 1 | 1 | 1 | 1 | 29,334 | 30,973 |
| 7 | 1 | 2 | 2 | 1 | 1 | 1 | 29,469 | 31,337 |
| 8 | 1 | 1 | 1 | 1 | 1 | 2 | 28,444 | 28,430 |
| 9 | 1 | 2 | 1 | 1 | 2 | 2 | 26,237 | 27,614 |
| 10 | 1 | 3 | 2 | 1 | 1 | 1 | 26,635 | 26,605 |

Table S3: Number of clusters of each label, for varying number of clusters using GMM clustering, and the Calinski-Harabasz score for each clustering.

## DMAS vs. LMAS

LMAS, the piecewise-linear version of the score, is preferable to DMAS, the step-wise discrete version, when looking at individual children. Its continuity means that children who fail the same tasks at similar, but not identical, ages, will get similar scores. However, when analyzing the developmental delay of large subpopulations, DMAS yields similar results, while having the advantages of being simpler to compute and more readily explainable in layman terms. Supplementary figures S1-S3 depict the same analysis as those of figures 2-4, using DMAS instead of LMAS.


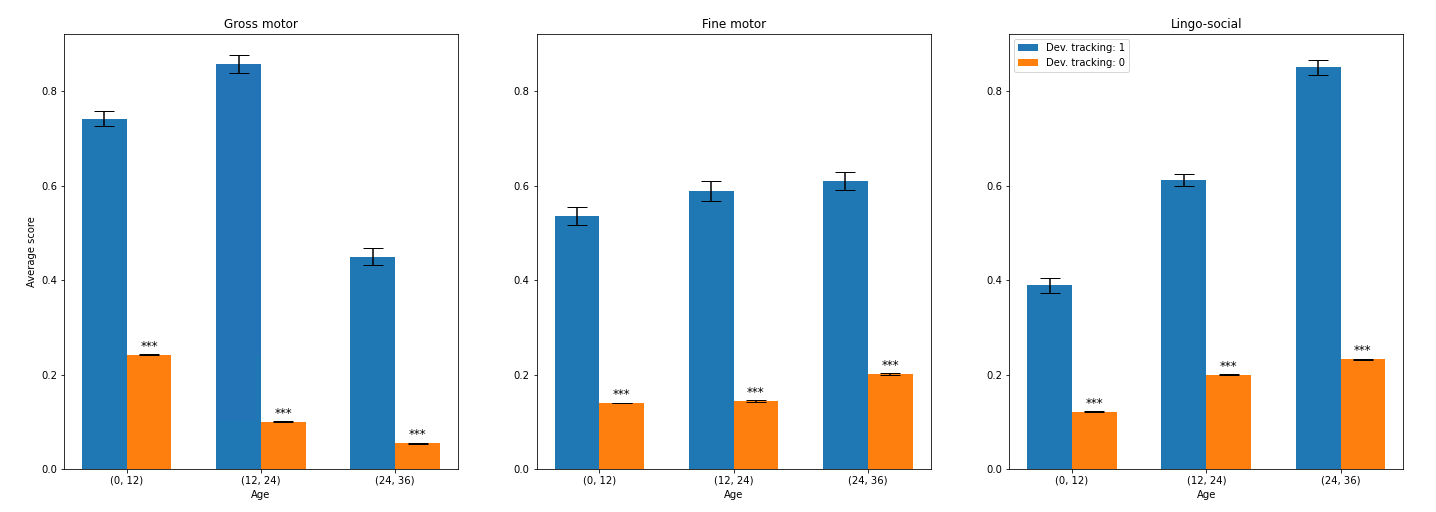
Figure S1a: DSS using DMAS of children who are under developmental tracking compared to those who are not. Asterixis denote columns which significantly differ from the ones to their left (* - p<0.05; ** - p<0.01; *** - p<0.001), according to Mann-Whitney U test.


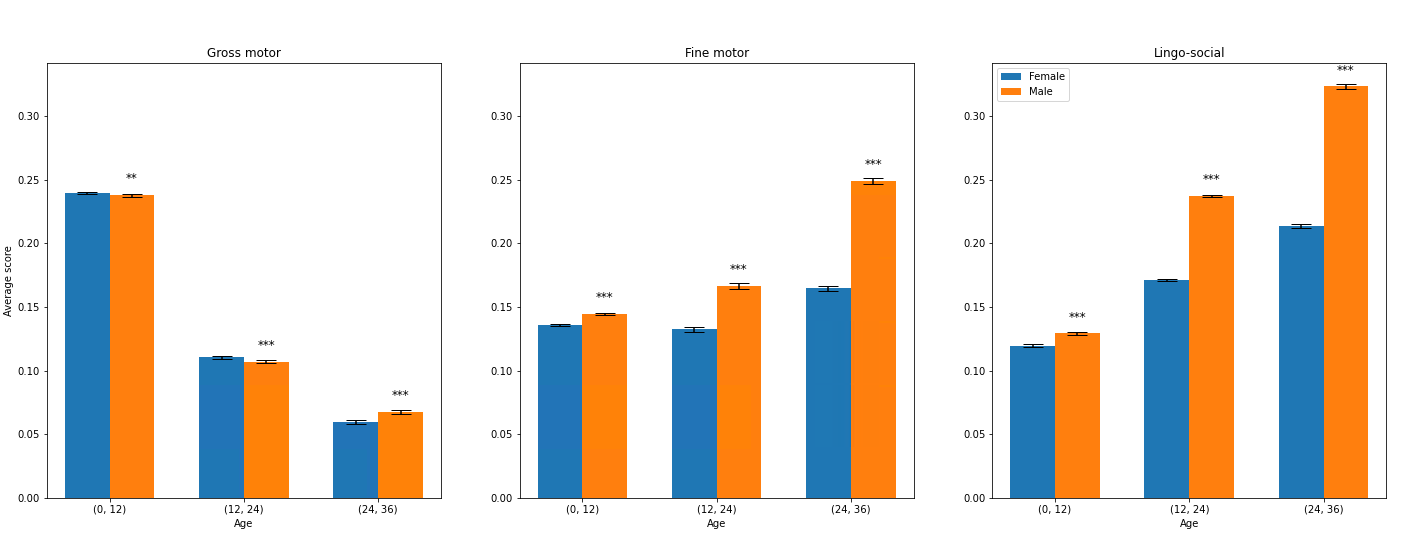
Figure S1b: DSS using DMAS of females compared to that of males.


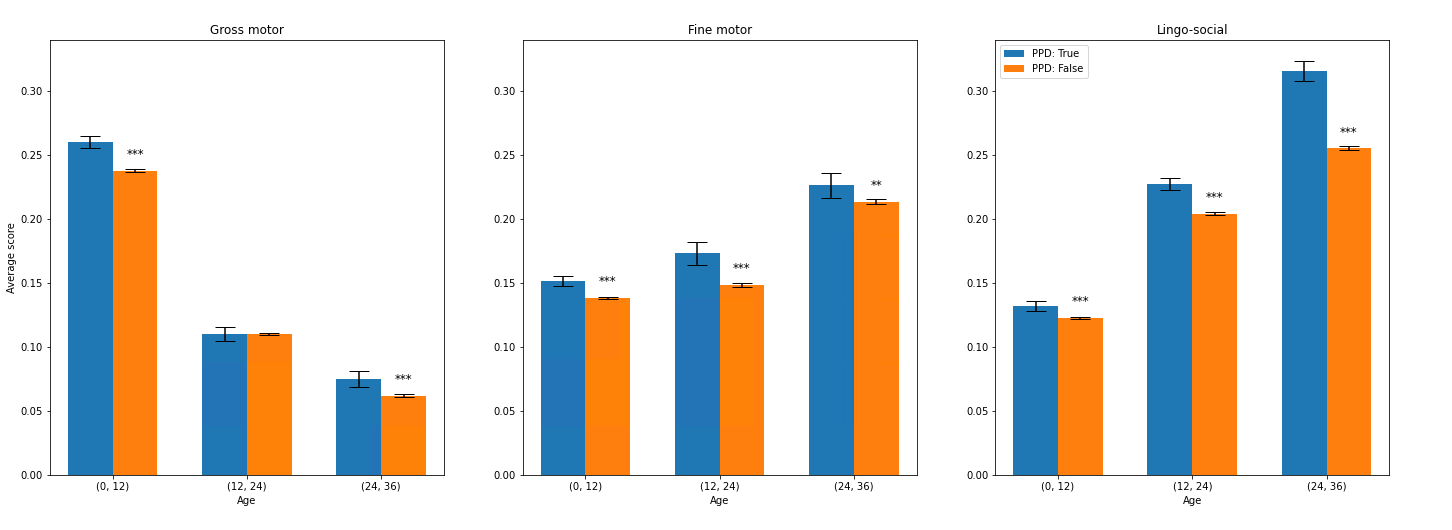
Figure S1c: DSS using DMAS of children whose mothers are suffering from PPD according to EPDS, compared to those who are not.


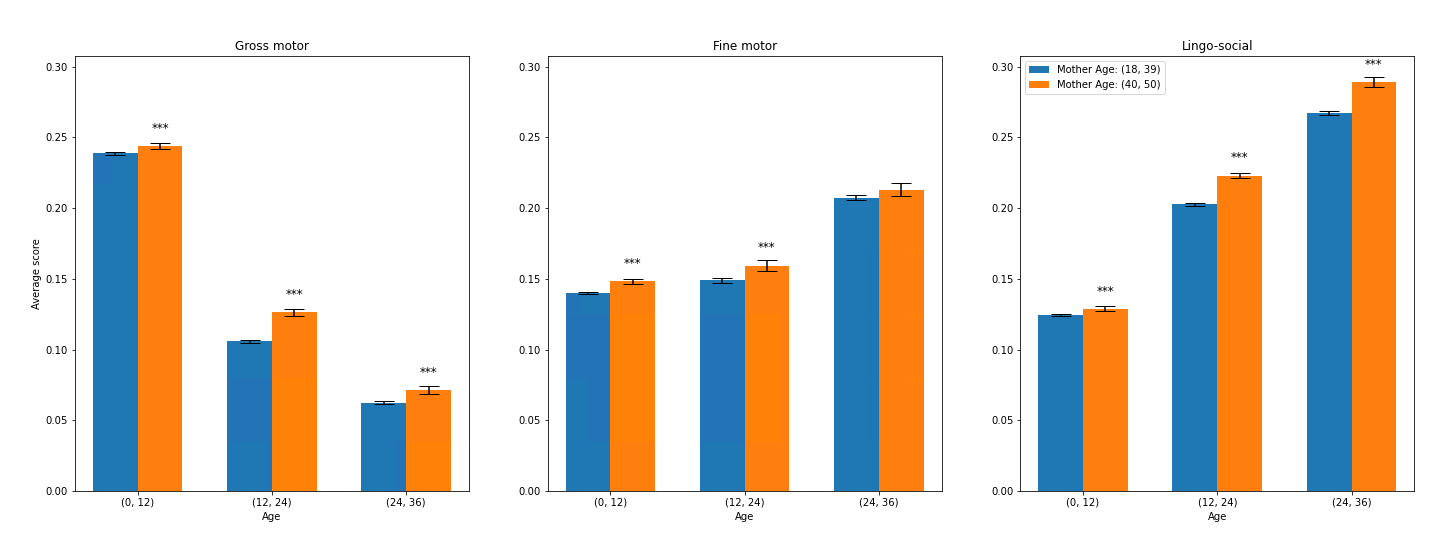
Figure S1d: DSS using DMAS of children of younger mothers (age 18-39) compared to those of older mothers (ages 40-50).


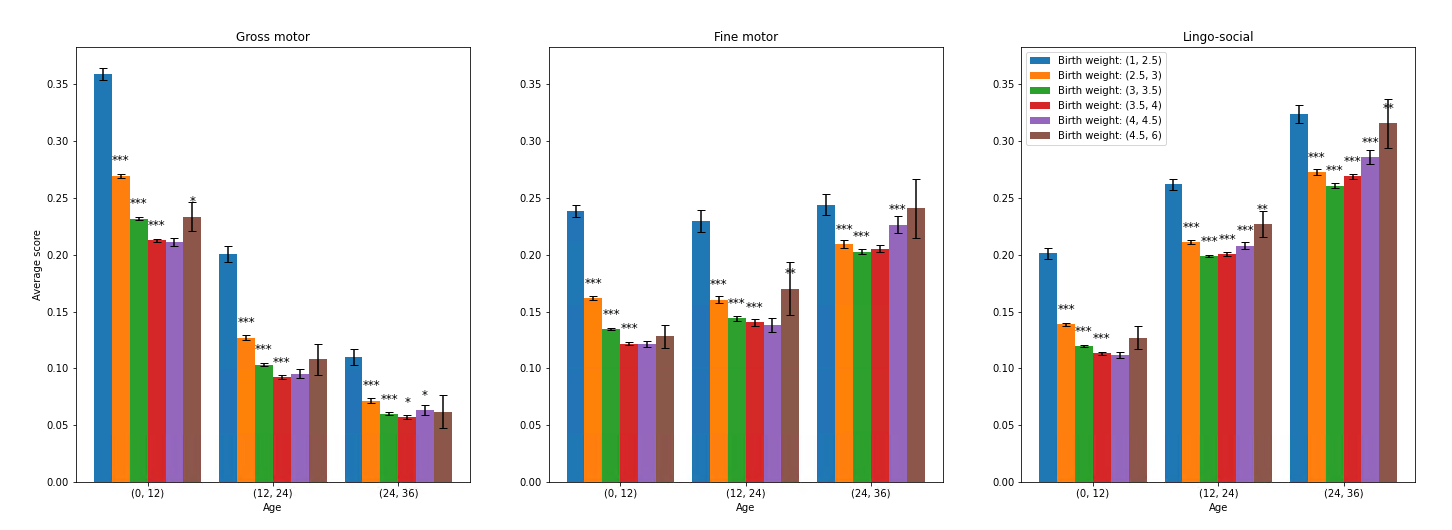
Figure S2a: Relation between DSS using DMAS and the child’s birth weight.


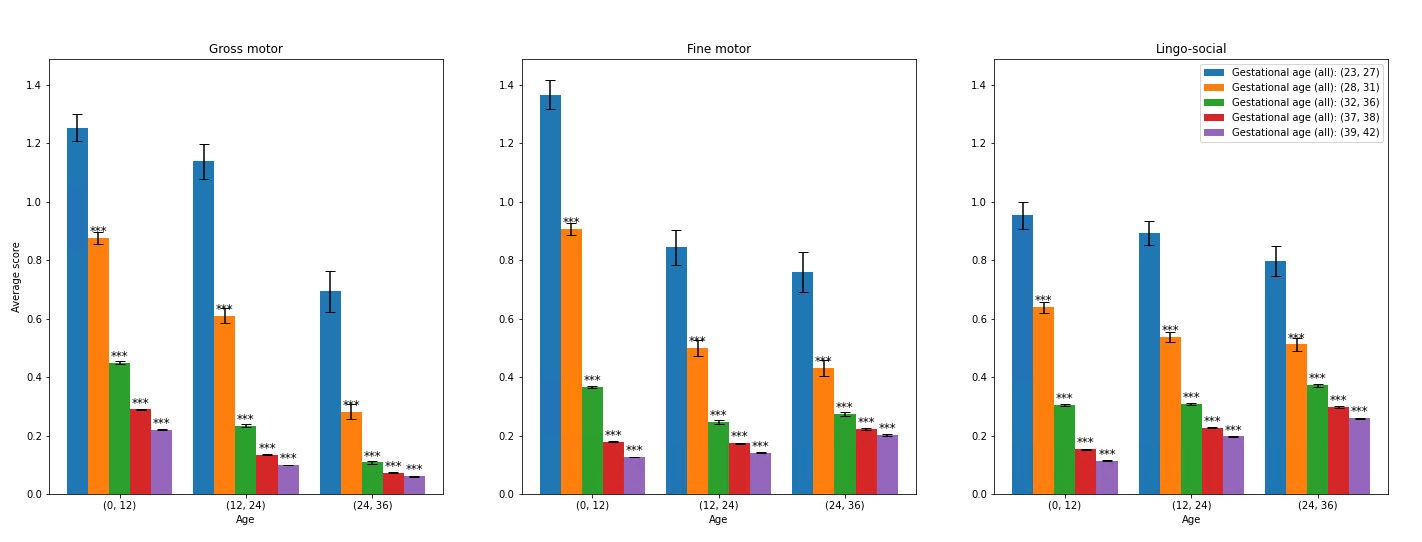
Figure S2b: Relation between DSS using DMAS and the gestational age.


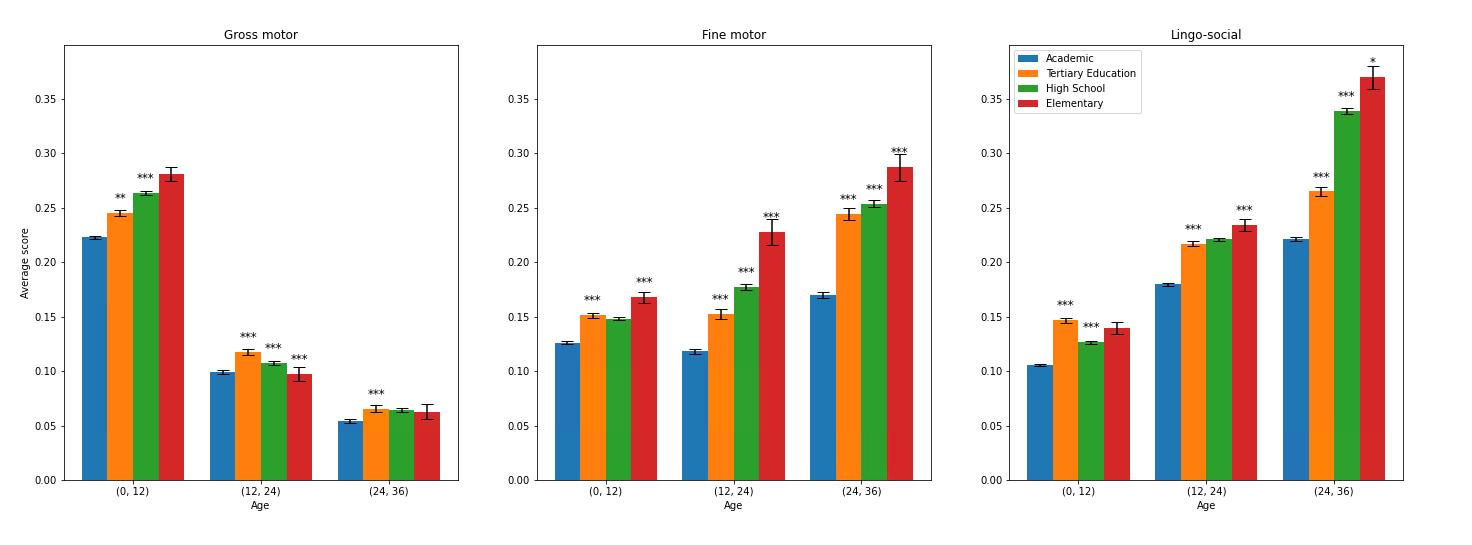
Figure S3a: Relation between DSS using DMAS and the the maternal education level.


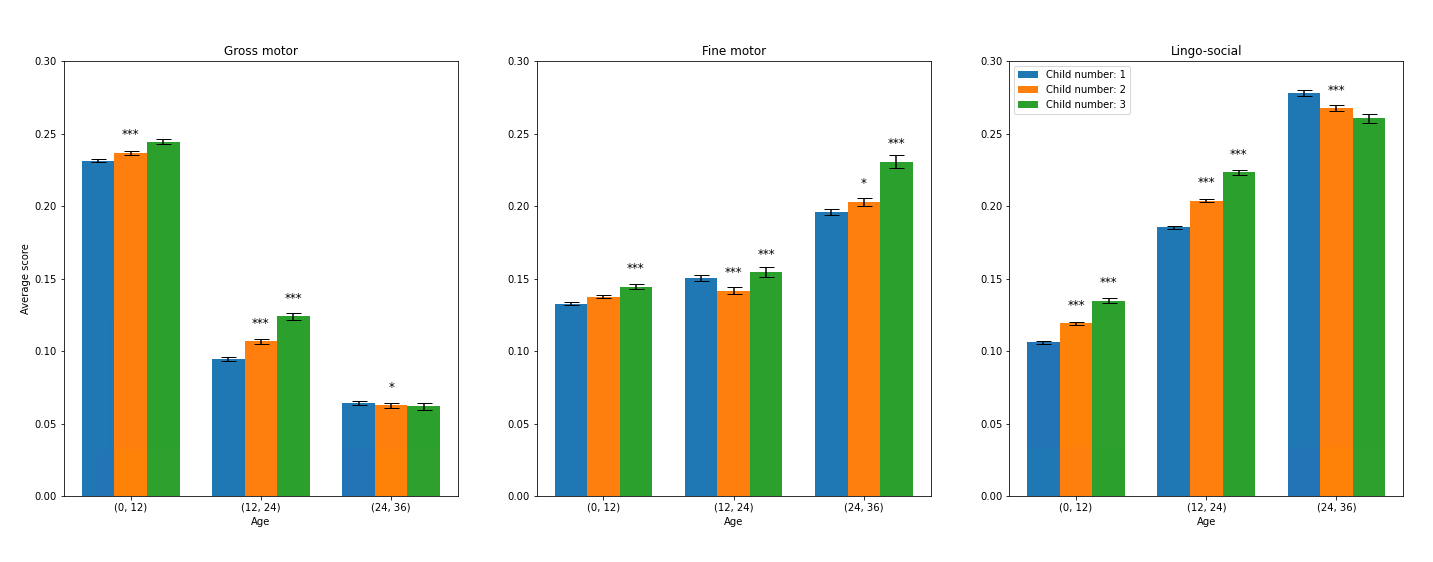
Figure S3b: Relation between DSS using DMAS and the child’s birth order.

## The q-score

Here we describe the “q-score”, an alternative to the DSS presented in the main paper. This score is motivated by the notion of Developmental Quotient (DQ) – the ratio between a child’s developmental age and chronological age.

For each milestone *m*, Sudry et al. [9] have estimated from the MCHC data of developmental assessments, a probability function, *P_m_(a)* that gives the probability for a neurotypical child at age *a* to attain milestone *m*. Accordingly, for a set of milestones, *m_1_,…, m_k_* attempted at ages *a_1_,…, a_k_*, the probability of attaining binary outcomes o_1_,…, o_k_ (where 1 indicates success and 0 indicates failure), assuming such events are independent, is:

$$\prod_{i} \left( P_{m_{i}}\left( a_{i} \right) \right)^{o_{i}}\left( 1-P_{m_{i}}\left( a_{i} \right) \right)^{1-o_{i}}$$

Now, suppose that a child who attempts these milestones at these ages has a developmental delay such that the ratio between their developmental age and chronological delay is *q* (when attempting any of these milestones). The probability for such a child to attain these outcomes is thus:

$$\prod_{i} \left( P_{m_{i}}\left( q\cdot a_{i} \right) \right)^{o_{i}}\left( 1-P_{m_{i}}\left( q\cdot a_{i} \right) \right)^{1-o_{i}}$$

Conversely, given a set of milestones, *m_1_,…, m_k_* attempted at ages *a_1_,…, a_k_* with outcomes o_1_,…, o_k_ we can compute the value of q for which the above expression best fits the empirical data. This is the q-score – the maximum likelihood estimator for the observed successes and failures in attaining a set of milestones. Note that rather than simply looking at P_m_(*a*), one can take into account additional information about the child, such as sex or gestational age. Hence one can estimate P_m_(*a*|*x*), where x is a vector of relevant covariates (e.g. using logistic regression), and plug that into the expression above.

The advantages of this formulation are that the q-score has a clear interpretation (it’s the ratio between developmental and chronological age), and it is derived in a formal mathematical way, rather than being based on the somewhat arbitrary thresholds applied in DSS. On the other hand, it relies on two strong assumptions – that success and failure at different milestones is independent, and that *q* is constant for the milestones and age ranges in question. Moreover, the derivation process might be more difficult to explain to someone lacking the relevant background, and is more difficult to compute.

A natural question is whether these two approaches lead to different conclusions when applied on real data. Namely, if we order children according to these scores, how different will the two orders be?

To test this, we considered children who were born on term (after July 1^st^ 2014), and their MCHC visits between ages 150-400 days. For each milestone we constructed a logistic regression model that estimates the probability of success given the age and sex of a child. For each child and for each developmental domain, we looked at all milestones of that domain during these visits and computed both DSS and q-score. Children who were not assessed in a specific domain were excluded from the analysis of this domain. This yielded cohorts of size 749,181 children for language-social milestones, 749,163 for gross motor milestones, and 738,312 for fine motor milestones. The agreement between the scores was assessed by computing them for each child, and then computing the Spearman rank correlation between the two lists of scores.

Table S4 suggests that the two scores, though derived in different ways, tend to agree on the ranking. Spearman rank correlation is 0.92 and higher for all three types of developmental domains. This is perhaps not surprising, since most children tend to attain all milestones, and so have a DMAS of 0, and a q-score of 1. Yet, even when we restrict the analysis to children with q-score<1 (33%,29% and 22% of the children for language-social, gross motor and fine-motor milestones, respectively) - the rank correlation remains high.

When DMAS is 0 (all milestones attained), the q-score is always 1. The converse is usually true, but not always. Specifically, when the q-score is 1, DMAS is 0 for 96.6% of children for language-social milestones, 98.8% for gross motor milestones and 99.9% for fine motor milestones. To see why this is not always the case, consider a child who attains all milestones but one, and fails that one milestone at an age where only 75% of children attain it. DMAS would be 1 (and LMAS between 0 and 1), yet the q-score would generally be 1 if the number of attained milestones is not very small.

| Cohort | language-social | Gross motor | Fine motor |
| --- | --- | --- | --- |
| All | 0.94 | 0.98 | 0.92 |
| q-score in (0,1) | 0.94 | 0.95 | 0.88 |

Table S4: Spearman rank correlation between LMAS and (the negative of) q-score, for all children and for children with q-score strictly between 0 and 1.
